# The complete mitochondrial genome analysis of *Eristalis tenax* (Diptera, Syrphidae)

**DOI:** 10.1080/23802359.2017.1375875

**Published:** 2017-09-11

**Authors:** Xiaoli Li, Shuangmei Ding, Xin Li, Peng Hou, Chufei Tang, Ding Yang

**Affiliations:** a College of Plant Protection, China Agricultural University, Beijing, China;; b College of Life Science, Shenyang Normal University, Shenyang, China

**Keywords:** Syrphidae, *Eristalis tenax*, mitochondrial genome, phylogeny

## Abstract

The complete mitochondrial genome of *Eristalis tenax* (Linnaeus, 1758) (Diptera, Syrphidae) is reported here. This is the first sequenced mitogenome from the subfamily Milesiinae. The whole mitochondrial genome is 16,091 bp in length and contains 37 canonical genes, which include 22 transfer RNA genes, 13 protein-coding genes and two ribosomal RNA genes, the control region is 1125 bp in length. Most PCGs start with standard ATN codons, while *CO1* and *ND1* use TTG, *CO3* uses TGG as start codons. All PCGs terminate in the common stop codons TAA. In addition, the nucleotide composition of the coding region was 40.0% of A, 40.1% of T, 11.2% of C, 8.7% of G and 80.1% of A + T content. The phylogenetic tree shows that Syrphidae is the sister group of Pipunculidae.

## Introduction

Syrphidae is one of the largest families of Diptera with nearly 6000 species widely distributed in the world (Sommaggio 1999; Zorica et al. 2013). The flower flies are ecologically important due to acting as plant pollination, predation of plant pests, phytophagy and nutrient recycling (Rotheray and Gilbert 2011). However, there are few mitochondrial genome data of Syrphidae. The mitochondrial genomes of its relative species *Platypeza* sp., *Lonchoptera multiseta* and *Pipunculus* sp. are available in our laboratory; *Megaselia scalaris* and *Simosyrphus grandicornis* are available in GenBank database. Hence, we sequenced the complete mitochondrial genome of *Eristalis tenax* (Linnaeus, 1758), the first representative species of subfamily Milesiinae for further research.

The specimen of *Eristalis tenax* used for DNA extraction was collected by Dr Yuqiang Xi from Xiaolongmen, Beijing, China, and identified by Dr Junchao Wang. After collection, the specimen was initially preserved in 95% ethanol in the field, and then transferred to −20 °C for the long-term storage after arrived at China Agricultural University. Specimens are deposited in the Entomological Museum of China Agricultural University, Beijing.

The whole genomic DNA was extracted from the thoracic muscle tissues using TIANamp Genomic DNA Kit (TIANGEN, Beijing, China), and sequenced under the next generation sequence technology.

The mitochondrial genome of *Eristalis tenax* contains all 37 genes, which include 22 transfer RNA genes, 13 protein-coding genes (PCGs), two ribosomal RNA genes and non-coding control region, and were similar with related reports before (Kang et al. 2014; Li et al. 2016; Wang, Ding, et al. 2016; Wang, Wang, et al. 2016, Wang, Liu, et al. 2016; Li et al. 2017; Zhou et al. 2017). Twenty-three genes of all genes were encoded by the majority strand (J-strand) and the other fourteen genes were encoded by the minority strand (N-strand). In this genome, we also found six overlaps ranging from 1 to 7 bp and 20 intergenic spacers whose sizes were between 1 and 37 bp except the control region between genes.

The base composition of the genome was 40.0% of A, 40.1% of T, 11.2% of C, 8.7% of G and 80.1% of A + T content. The 10 of 13 PCGs use the normal ATN start codons, while the *CO1* and *ND1* use TTG, the *CO3* uses TGG as start codons. For termination codons, all PCGs use TAA as stop codons.

**Figure 1.**
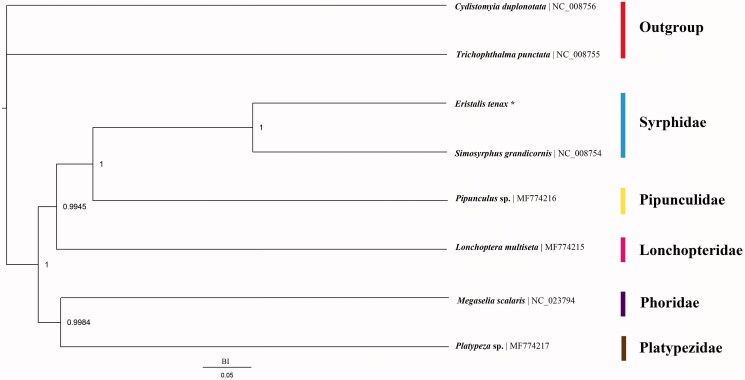
Phylogenetic tree among eight species which consist of six lower Cyclorrhapha species and two outgroups Nemestrinidae and Tabanidae (*represent data sequenced in this study).

A total of eight species were used in phylogenetic analysis, including two outgroup species from Nemestrinidae and Tabanidae. Details of the species used in this study are listed in Figure 1. The phylogenetic tree from Bayesian (BI) analyses using dataset contains all PCGs genes which has 11,136 residues (Ronquist and Huelsenbeck 2003) (Figure 1). The result supports the monophyly of Syrphidae, and shows that Syrphidae is the sister group of Pipunculidae.
